# (*E*)-*N*′-(2-Fluoro­benzyl­idene)furan-2-carbohydrazide

**DOI:** 10.1107/S1600536810052980

**Published:** 2011-01-08

**Authors:** Jin-He Jiang

**Affiliations:** a Microscale Science Institute, Department of Chemistry and Chemical Engineering, Weifang University, Weifang 261061, People’s Republic of China

## Abstract

The title compound, C_12_H_9_FN_2_O_2_, was prepared by the reaction of 2-fluoro­benzaldehyde and furan-2-carbohydrazide. The furan ring is disordered over two sets of sites with refined occupancies of 0.60 (3):0.40 (3). The major and minor components of the furan ring make dihedral angles of 51.9 (6) and 38.0 (10)°, respectively, with the benzene ring. In the crystal, mol­ecules are linked *via* bifurcated N—H⋯O(N) hydrogen bonds into chains along [001].

## Related literature

For related structures, see: Li & Jian (2010 ▶); Li & Meng (2010 ▶).
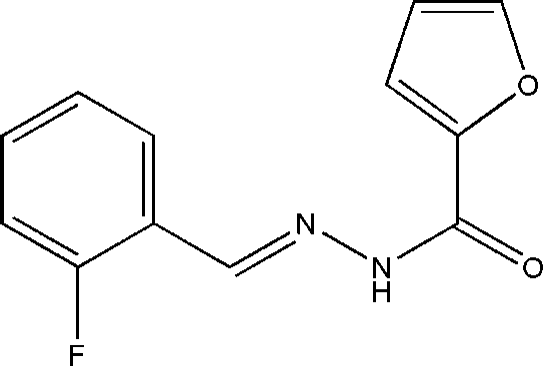


## Experimental

### 

#### Crystal data

C_12_H_9_FN_2_O_2_
*M* *_r_* = 232.21
Monoclinic, 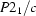

*a* = 11.719 (2) Å
*b* = 13.395 (3) Å
*c* = 7.5154 (15) Å
β = 105.04 (3)°
*V* = 1139.3 (4) Å^3^
*Z* = 4
Mo *K*α radiation
μ = 0.11 mm^−1^
*T* = 293 K
0.23 × 0.19 × 0.18 mm

#### Data collection

Bruker SMART CCD diffractometer
10898 measured reflections
2597 independent reflections
1341 reflections with *I* > 2σ(*I*)
*R*_int_ = 0.040

#### Refinement

*R*[*F* ^2^ > 2σ(*F* ^2^)] = 0.043
*wR*(*F* ^2^) = 0.158
*S* = 1.12
2597 reflections
200 parameters
39 restraints
H-atom parameters constrained
Δρ_max_ = 0.18 e Å^−3^
Δρ_min_ = −0.20 e Å^−3^

### 

Data collection: *SMART* (Bruker, 1997 ▶); cell refinement: *SAINT* (Bruker, 1997 ▶); data reduction: *SAINT*; program(s) used to solve structure: *SHELXS97* (Sheldrick, 2008 ▶); program(s) used to refine structure: *SHELXL97* (Sheldrick, 2008 ▶); molecular graphics: *SHELXTL* (Sheldrick, 2008 ▶); software used to prepare material for publication: *SHELXTL*.

## Supplementary Material

Crystal structure: contains datablocks global, I. DOI: 10.1107/S1600536810052980/lh5184sup1.cif

Structure factors: contains datablocks I. DOI: 10.1107/S1600536810052980/lh5184Isup2.hkl

Additional supplementary materials:  crystallographic information; 3D view; checkCIF report

## Figures and Tables

**Table 1:** Hydrogen-bond geometry (Å, °)

*D*—H⋯*A*	*D*—H	H⋯*A*	*D*⋯*A*	*D*—H⋯*A*
N1—H1⋯O2^i^	0.86	2.13	2.956 (2)	162
N1—H1⋯N2^i^	0.86	2.63	3.216 (3)	127

Symmetry code: (i) 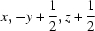.

**Fig. 1.**
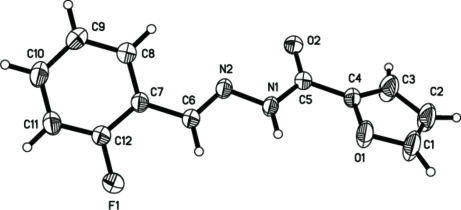
The molecular structure of the title compound showing 30% probability displacement ellipsoids and the atom-numbering scheme. The disorder is not shown.

## supplementary crystallographic information

### Comment

The molecular structure of the title compound is shown in Fig. 1. The bond lengths and angles in the carbohydrazide group of the title compound can be compared with two examples of thiosemicarbazides recently published (Li & Jian, 2010; Li & Meng,2010). In the title molecule, the furan ring is disordered over two sets of sites with refined occupancies of 0.60 (3):0.40 (3). The diherdal angles that the major and minor components of the furan ring make with the benzene ring are 51.9 (6) and 38.0 (10)°, respectively. In the crystal, molecules are linked via bifurcated N—H···O(N) hydrogen bonds to form one-dimensional chains along [001].

### Experimental

A mixture of 2-fluorobenzaldehyde (0.01 mol) and furan-2-carbohydrazide (0.01 mol) was stirred in refluxing ethanol (20 mL) for 2h to afford the title compound (0.087 mol, yield 87%). Single crystals suitable for X-ray measurements were obtained by recrystallization of a solution of the title compound in ethanol at room temperature.

### Refinement

H atoms were fixed geometrically and allowed to ride on their attached atoms, with C—H  = 0.93 Å; N—H = 0.86Å and with U_iso_(H) = 1.2_eq_(C, N).

### Crystal data

**Table:**

C_12_H_9_FN_2_O_2_	*F*(000) = 480
*M**_r_* = 232.21	*D*_x_ = 1.354 Mg m^−^^3^
Monoclinic, *P*2_1_/*c*	Mo *K*α radiation, λ = 0.71073 Å
Hall symbol: -P 2ybc	Cell parameters from 2597 reflections
*a* = 11.719 (2) Å	θ = 3.0–27.5°
*b* = 13.395 (3) Å	µ = 0.11 mm^−^^1^
*c* = 7.5154 (15) Å	*T* = 293 K
β = 105.04 (3)°	Bar, colorless
*V* = 1139.3 (4) Å^3^	0.23 × 0.19 × 0.18 mm
*Z* = 4	

### Data collection

**Table:**

Bruker SMART CCD diffractometer	1341 reflections with *I* > 2σ(*I*)
Radiation source: fine-focus sealed tube	*R*_int_ = 0.040
graphite	θ_max_ = 27.5°, θ_min_ = 3.0°
φ and ω scans	*h* = −15→15
10898 measured reflections	*k* = −15→17
2597 independent reflections	*l* = −9→9

### Refinement

**Table:**

Refinement on *F*^2^	Primary atom site location: structure-invariant direct methods
Least-squares matrix: full	Secondary atom site location: difference Fourier map
*R*[*F*^2^ > 2σ(*F*^2^)] = 0.043	Hydrogen site location: inferred from neighbouring sites
*wR*(*F*^2^) = 0.158	H-atom parameters constrained
*S* = 1.12	*w* = 1/[σ^2^(*F*_o_^2^) + (0.0717*P*)^2^ + 0.0765*P*] where *P* = (*F*_o_^2^ + 2*F*_c_^2^)/3
2597 reflections	(Δ/σ)_max_ < 0.001
200 parameters	Δρ_max_ = 0.18 e Å^−^^3^
39 restraints	Δρ_min_ = −0.20 e Å^−^^3^

### Special details

**Table:**

Geometry. All e.s.d.'s (except the e.s.d. in the dihedral angle between two l.s. planes) are estimated using the full covariance matrix. The cell e.s.d.'s are taken into account individually in the estimation of e.s.d.'s in distances, angles and torsion angles; correlations between e.s.d.'s in cell parameters are only used when they are defined by crystal symmetry. An approximate (isotropic) treatment of cell e.s.d.'s is used for estimating e.s.d.'s involving l.s. planes.
Refinement. Refinement of *F*^2^ against ALL reflections. The weighted *R*-factor *wR* and goodness of fit *S* are based on *F*^2^, conventional *R*-factors *R* are based on *F*, with *F* set to zero for negative *F*^2^. The threshold expression of *F*^2^ > σ(*F*^2^) is used only for calculating *R*-factors(gt) *etc*. and is not relevant to the choice of reflections for refinement. *R*-factors based on *F*^2^ are statistically about twice as large as those based on *F*, and *R*- factors based on ALL data will be even larger.

### Fractional atomic coordinates and isotropic or equivalent isotropic displacement parameters (Å^2^)

**Table:**

	*x*	*y*	*z*	*U*_iso_*/*U*_eq_	Occ. (<1)
F1	0.43175 (12)	0.53700 (8)	0.31687 (19)	0.0719 (5)	
O1	0.0418 (10)	0.1625 (9)	0.4392 (11)	0.081 (2)	0.60 (3)
C1	−0.0584 (13)	0.1286 (11)	0.4755 (19)	0.115 (5)	0.60 (3)
H1A	−0.0803	0.1413	0.5837	0.138*	0.60 (3)
C2	−0.1205 (12)	0.0754 (13)	0.338 (2)	0.106 (5)	0.60 (3)
H2A	−0.1905	0.0417	0.3340	0.128*	0.60 (3)
C3	−0.0611 (9)	0.0790 (11)	0.1994 (16)	0.094 (4)	0.60 (3)
H3A	−0.0861	0.0508	0.0826	0.112*	0.60 (3)
C4	0.0386 (10)	0.1308 (12)	0.2665 (16)	0.049 (3)	0.60 (3)
C5	0.13101 (18)	0.16408 (13)	0.1818 (3)	0.0467 (5)	
O1A	0.0064 (15)	0.1954 (17)	0.388 (3)	0.091 (4)	0.40 (3)
C1A	−0.0962 (19)	0.161 (2)	0.414 (4)	0.136 (9)	0.40 (3)
H1AA	−0.1274	0.1796	0.5112	0.163*	0.40 (3)
C2A	−0.1460 (15)	0.097 (2)	0.286 (3)	0.109 (7)	0.40 (3)
H2AA	−0.2194	0.0666	0.2704	0.131*	0.40 (3)
C3A	−0.0675 (10)	0.0831 (13)	0.176 (2)	0.080 (4)	0.40 (3)
H3AA	−0.0755	0.0378	0.0800	0.096*	0.40 (3)
C4A	0.0216 (14)	0.1477 (18)	0.236 (3)	0.051 (4)	0.40 (3)
O2	0.14506 (13)	0.12419 (9)	0.04193 (19)	0.0578 (4)	
N1	0.20365 (15)	0.23571 (11)	0.2747 (2)	0.0485 (4)	
H1	0.1890	0.2647	0.3684	0.058*	
N2	0.30170 (15)	0.26118 (11)	0.2161 (2)	0.0473 (4)	
C6	0.35441 (18)	0.34096 (13)	0.2850 (3)	0.0476 (5)	
H6A	0.3232	0.3800	0.3633	0.057*	
C7	0.46353 (18)	0.37102 (13)	0.2411 (3)	0.0473 (5)	
C8	0.5360 (2)	0.30396 (16)	0.1816 (3)	0.0646 (6)	
H8A	0.5138	0.2373	0.1648	0.078*	
C9	0.6401 (2)	0.33467 (18)	0.1471 (4)	0.0802 (8)	
H9A	0.6872	0.2890	0.1057	0.096*	
C10	0.6750 (2)	0.43347 (19)	0.1738 (4)	0.0784 (8)	
H10A	0.7464	0.4537	0.1528	0.094*	
C11	0.6051 (2)	0.50132 (18)	0.2309 (3)	0.0654 (7)	
H11A	0.6275	0.5680	0.2481	0.078*	
C12	0.50190 (19)	0.46913 (14)	0.2619 (3)	0.0521 (5)	

### Atomic displacement parameters (Å^2^)

**Table:**

	*U*^11^	*U*^22^	*U*^33^	*U*^12^	*U*^13^	*U*^23^
F1	0.0725 (10)	0.0524 (7)	0.0988 (10)	−0.0030 (6)	0.0365 (8)	−0.0100 (6)
O1	0.089 (4)	0.101 (4)	0.067 (3)	−0.054 (3)	0.044 (3)	−0.025 (3)
C1	0.126 (7)	0.143 (7)	0.106 (6)	−0.090 (7)	0.083 (6)	−0.054 (6)
C2	0.101 (7)	0.124 (8)	0.116 (6)	−0.073 (8)	0.068 (6)	−0.047 (6)
C3	0.080 (6)	0.124 (8)	0.088 (4)	−0.057 (6)	0.042 (4)	−0.043 (5)
C4	0.049 (3)	0.049 (4)	0.054 (3)	−0.009 (4)	0.020 (3)	−0.003 (4)
C5	0.0477 (11)	0.0443 (10)	0.0512 (11)	−0.0034 (8)	0.0184 (9)	−0.0013 (9)
O1A	0.074 (6)	0.124 (8)	0.092 (6)	−0.043 (6)	0.054 (6)	−0.043 (6)
C1A	0.089 (9)	0.182 (17)	0.170 (15)	−0.061 (10)	0.094 (11)	−0.076 (13)
C2A	0.055 (6)	0.116 (10)	0.170 (16)	−0.031 (6)	0.054 (8)	−0.036 (10)
C3A	0.058 (6)	0.060 (7)	0.129 (9)	−0.019 (5)	0.036 (6)	−0.028 (6)
C4A	0.044 (4)	0.051 (7)	0.060 (6)	−0.007 (5)	0.017 (5)	−0.003 (6)
C5A	0.0477 (11)	0.0443 (10)	0.0512 (11)	−0.0034 (8)	0.0184 (9)	−0.0013 (9)
O2	0.0627 (11)	0.0534 (8)	0.0635 (10)	−0.0095 (7)	0.0273 (8)	−0.0141 (7)
N1	0.0484 (11)	0.0552 (9)	0.0479 (9)	−0.0149 (7)	0.0232 (8)	−0.0077 (7)
N2	0.0439 (10)	0.0513 (9)	0.0506 (9)	−0.0080 (7)	0.0193 (8)	−0.0005 (7)
C6	0.0486 (13)	0.0471 (10)	0.0512 (12)	−0.0050 (9)	0.0203 (10)	−0.0020 (8)
C7	0.0460 (12)	0.0492 (10)	0.0495 (12)	−0.0070 (8)	0.0173 (10)	−0.0012 (8)
C8	0.0607 (15)	0.0562 (12)	0.0855 (17)	−0.0086 (11)	0.0343 (13)	−0.0112 (11)
C9	0.0643 (17)	0.0822 (16)	0.108 (2)	−0.0072 (13)	0.0475 (16)	−0.0231 (15)
C10	0.0602 (17)	0.0895 (17)	0.0962 (19)	−0.0231 (13)	0.0397 (15)	−0.0146 (14)
C11	0.0638 (16)	0.0630 (13)	0.0750 (16)	−0.0211 (11)	0.0281 (13)	−0.0075 (11)
C12	0.0526 (14)	0.0514 (11)	0.0559 (12)	−0.0053 (9)	0.0207 (11)	−0.0051 (9)

### Geometric parameters (Å, °)

**Table:**

F1—C12	1.360 (2)	C3A—C4A	1.341 (4)
O1—C1	1.351 (4)	C3A—H3AA	0.9300
O1—C4	1.357 (4)	N1—N2	1.376 (2)
C1—C2	1.308 (5)	N1—H1	0.8600
C1—H1A	0.9300	N2—C6	1.275 (2)
C2—C3	1.397 (6)	C6—C7	1.458 (3)
C2—H2A	0.9300	C6—H6A	0.9300
C3—C4	1.340 (4)	C7—C12	1.385 (3)
C3—H3A	0.9300	C7—C8	1.387 (3)
C4—C5	1.461 (4)	C8—C9	1.375 (3)
C5—O2	1.228 (2)	C8—H8A	0.9300
C5—N1	1.351 (2)	C9—C10	1.384 (3)
O1A—C1A	1.351 (4)	C9—H9A	0.9300
O1A—C4A	1.357 (4)	C10—C11	1.365 (3)
C1A—C2A	1.308 (6)	C10—H10A	0.9300
C1A—H1AA	0.9300	C11—C12	1.360 (3)
C2A—C3A	1.397 (6)	C11—H11A	0.9300
C2A—H2AA	0.9300		
			
C1—O1—C4	106.1 (3)	C3A—C4A—O1A	109.1 (3)
C2—C1—O1	111.0 (3)	C5—N1—N2	118.48 (16)
C2—C1—H1A	124.5	C5—N1—H1	120.8
O1—C1—H1A	124.5	N2—N1—H1	120.8
C1—C2—C3	106.7 (3)	C6—N2—N1	115.75 (16)
C1—C2—H2A	126.7	N2—C6—C7	120.04 (17)
C3—C2—H2A	126.7	N2—C6—H6A	120.0
C4—C3—C2	106.9 (4)	C7—C6—H6A	120.0
C4—C3—H3A	126.5	C12—C7—C8	116.29 (19)
C2—C3—H3A	126.5	C12—C7—C6	120.98 (17)
C3—C4—O1	109.1 (3)	C8—C7—C6	122.70 (17)
C3—C4—C5	131.8 (5)	C9—C8—C7	121.0 (2)
O1—C4—C5	118.8 (2)	C9—C8—H8A	119.5
O2—C5—N1	123.05 (18)	C7—C8—H8A	119.5
O2—C5—C4	121.3 (3)	C8—C9—C10	120.1 (2)
N1—C5—C4	115.5 (2)	C8—C9—H9A	119.9
C1A—O1A—C4A	106.1 (3)	C10—C9—H9A	119.9
C2A—C1A—O1A	111.1 (3)	C11—C10—C9	120.2 (2)
C2A—C1A—H1AA	124.5	C11—C10—H10A	119.9
O1A—C1A—H1AA	124.5	C9—C10—H10A	119.9
C1A—C2A—C3A	106.6 (3)	C12—C11—C10	118.4 (2)
C1A—C2A—H2AA	126.7	C12—C11—H11A	120.8
C3A—C2A—H2AA	126.7	C10—C11—H11A	120.8
C4A—C3A—C2A	106.8 (4)	C11—C12—F1	118.34 (18)
C4A—C3A—H3AA	126.6	C11—C12—C7	123.97 (19)
C2A—C3A—H3AA	126.6	F1—C12—C7	117.69 (17)
			
C4—O1—C1—C2	2.1 (18)	C4—C5—N1—N2	171.6 (9)
O1—C1—C2—C3	−3(2)	C5—N1—N2—C6	167.22 (18)
C1—C2—C3—C4	3(2)	N1—N2—C6—C7	176.02 (17)
C2—C3—C4—O1	−2(2)	N2—C6—C7—C12	158.9 (2)
C2—C3—C4—C5	−175.4 (17)	N2—C6—C7—C8	−22.8 (3)
C1—O1—C4—C3	0.1 (18)	C12—C7—C8—C9	0.4 (4)
C1—O1—C4—C5	174.4 (13)	C6—C7—C8—C9	−177.9 (2)
C3—C4—C5—O2	−21 (3)	C7—C8—C9—C10	0.9 (4)
O1—C4—C5—O2	166.6 (10)	C8—C9—C10—C11	−1.4 (4)
C3—C4—C5—N1	163.9 (18)	C9—C10—C11—C12	0.6 (4)
O1—C4—C5—N1	−8.8 (17)	C10—C11—C12—F1	−179.3 (2)
C4A—O1A—C1A—C2A	−2(3)	C10—C11—C12—C7	0.7 (4)
O1A—C1A—C2A—C3A	5(3)	C8—C7—C12—C11	−1.2 (3)
C1A—C2A—C3A—C4A	−6(3)	C6—C7—C12—C11	177.1 (2)
C2A—C3A—C4A—O1A	5(3)	C8—C7—C12—F1	178.85 (19)
C1A—O1A—C4A—C3A	−2(3)	C6—C7—C12—F1	−2.8 (3)
O2—C5—N1—N2	−3.8 (3)		

### Hydrogen-bond geometry (Å, °)

**Table:**

*D*—H···*A*	*D*—H	H···*A*	*D*···*A*	*D*—H···*A*
N1—H1···O2^i^	0.86	2.13	2.956 (2)	162.
N1—H1···N2^i^	0.86	2.63	3.216 (3)	127.

Symmetry codes: (i) *x*, −*y*+1/2, *z*+1/2.
